# Hierarchical Hidden Markov models enable accurate and diverse detection of antimicrobial resistance sequences

**DOI:** 10.1038/s42003-019-0545-9

**Published:** 2019-08-06

**Authors:** Steven M. Lakin, Alan Kuhnle, Bahar Alipanahi, Noelle R. Noyes, Chris Dean, Martin Muggli, Rob Raymond, Zaid Abdo, Mattia Prosperi, Keith E. Belk, Paul S. Morley, Christina Boucher

**Affiliations:** 1 Department of Microbiology, Immunology, and Pathology, Colorado State University, Fort Collins, CO 80523 USA; 2 Department of Computer and Information Science and Engineering, University of Florida, Gainesville, FL 32611 USA; 3 Department of Veterinary Population Medicine, University of Minnesota, St. Paul, MN 55108 USA; 4 Department of Computer Science, Colorado State University, Fort Collins, CO 80523 USA; 5 Department of Epidemiology, University of Florida, Gainesville, FL 32611 USA; 6 Department of Animal Sciences, Colorado State University, Fort Collins, CO 80523 USA; 7 VERO Center, Texas A&M University and West Texas A&M University, Canyon, TX 79016 USA

**Keywords:** Microbial genetics, Machine learning, Microbial communities, Antimicrobial resistance

## Abstract

The characterization of antimicrobial resistance genes from high-throughput sequencing data has become foundational in public health research and regulation. This requires mapping sequence reads to databases of known antimicrobial resistance genes to determine the genes present in the sample. Mapping sequence reads to known genes is traditionally accomplished using alignment. Alignment methods have high specificity but are limited in their ability to detect sequences that are divergent from the reference database, which can result in a substantial false negative rate. We address this shortcoming through the creation of Meta-MARC, which enables detection of diverse resistance sequences using hierarchical, DNA-based Hidden Markov Models. We first describe Meta-MARC and then demonstrate its efficacy on simulated and functional metagenomic datasets. Meta-MARC has higher sensitivity relative to competing methods. This sensitivity allows for detection of sequences that are divergent from known antimicrobial resistance genes. This functionality is imperative to expanding existing antimicrobial gene databases.

## Abstract (web summary)

Steven Lakin et al. present Meta-MARC, a computational method for identifying antimicrobial resistance sequences using DNA-based Hidden Markov Models. Because of its increased sensitivity, Meta-MARC is able to detect novel antimicrobial resistance sequences that are divergent from all known sequences.

## Introduction

Antimicrobial resistance (AMR) continues to be a critical concern in medical treatment, environmental management, and food safety^1–5^. AMR in bacteria occurs either by mutation or acquisition of genes that circumvent or counteract an antimicrobial’s biological mechanism of action or decrease the concentration of antimicrobial compounds within bacterial cells. The collection of these AMR genes in both pathogenic and non-pathogenic microbes is commonly referred to as the resistome and defines a population’s potential resistance to known antimicrobials.

To better understand the relationship between antimicrobial use and the resistome, great effort has been spent on determining the global effects of antimicrobial use on resistome composition^6^, the retention of AMR genes in soil, food-production, and hospital environments^3,5,7–9^ and the molecular mechanisms of action and transmission of AMR genes^10^. As a result, the characterization of AMR has moved away from culture-dependent methods in favor of application and analysis of high-throughput sequence data, which allows for increased resolution in characterizing the microbiome and resistome—particularly in an ecological context^11^. Traditionally, analysis of high-throughput sequence data in this context involves mapping the sequence reads to databases of known AMR genes, then post-processing the alignment to predict which AMR genes are contained in the sample^12^. Short-read sequence aligners—such as the Burrows Wheeler Aligner (BWA)^13^ and Bowtie^14^—have most commonly been used for mapping the sequence reads. For example, AMRPlusPlus uses the Burrows Wheeler Aligner to align metagenomic sequence reads to the MEGARes database and classifies each read according to the gene to which it aligns^12^.

However, short-read aligners are only capable of identifying genes from sequence reads that differ by at most 10 or 12 nucleotides from the corresponding reference sequence^15^; therefore, sequence reads with even a small divergence from the reference remain unmapped and are eliminated from further analysis. This results in a high proportion of unmapped sequence data, and a high likelihood of false negatives. Recent studies related to microbiome genus-level taxonomic classification showed that upwards of 60% of sequence reads cannot be classified to known DNA reference sequences^16–18^ these results may similarly affect functional characterization (such as the resistome).

The low sensitivity of alignment-based methods for characterizing the resistome is especially problematic when analyzing high-throughput sequence datasets with low-read abundance; such datasets occur when shallow sequencing is performed, or when samples with a low abundance of DNA are investigated^19^. Machine learning classifiers present an opportunity for increasing the sensitivity of classification over alignment^20^, and thus have been previously used with high-throughput sequence data to characterize the resistome^21^. For example, Resfams uses Hidden Markov Models (HMMs) to classify AMR-related protein sequences from high-throughput sequence data by assembling the sequence reads using a standard genome assembler, translating the resulting contigs into amino acid sequences, and classifying these translated sequences^21^.

In this paper, we present Meta-MARC, which is a machine learning classifier that predicts the contents of the resistome for a given high-throughput sequence dataset from DNA sequences, removing the necessity of assembling or translating the data. Meta-MARC is constructed from the MEGARes database and is built by clustering all AMR genes based on sequence similarity and then training a model for each cluster. Using the hierarchical structure of MEGARes, the AMR Class, Group and Mechanism can be inferred from the sequence annotations. Thus, Meta-MARC predicts an AMR model for each input sequence, which corresponds to an AMR Class, Group(s), and Mechanism(s). Since Meta-MARC is developed and trained on a DNA AMR database, it eliminates the need for translation from DNA to amino acid sequence. We show that this advancement from existing machine learning classification methods (e.g., Resfams) provides a substantial increase in the percentage of classified high-throughput sequence data and the number of AMR classes identified.

We demonstrate the effectiveness of Meta-MARC on simulated, functional metagenomic, and shotgun metagenomic data by comparison with current state-of-the-art resistome classifiers and using robust validation. The results show that Meta-MARC has extremely high sensitivity and specificity on simulated data (≥98% sensitivity and ≥99% specificity), classifies a higher proportion of the high-throughput sequence data than competing methods, and is robust to genetic variation—making it invaluable for analysis of high-throughput sequence datasets with low-read abundance.

## Results

### Cross-validation of Meta-MARC using simulated data

To evaluate performance, we performed leave-one-out cross-validation^22^ on the Meta-MARC models that were constructed using more than two sequences. For each such model, we randomly selected a sequence from the set of sequences used to train the model, removed it from training set, and retrained the model. The removed sequence was added to the test set for that model, along with AMR sequences published in CARD between August 2016 and January 2017^23^ (See https://github.com/lakinsm/meta-marc-publication/blob/master/analytic_data/mmarc_test_set.fasta). These additional sequences were chosen because they were not included in the construction of the Meta-MARC HMMs but were annotated by AMR Class, Mechanism and Group. Next, 150 bp reads were simulated from the sequences in the test set. The sequence reads were simulated with a mean insert size of 250 bp and standard deviation of 10 bp at 2x coverage over the AMR gene with a HiSeq Illumina error profile using ART^24^. This simulation resulted in a total of 20–100 sequence reads for each left-out sequence (depending on its length), and 44,700 total sequence reads from CARD sequences. Each model was then tested with the corresponding simulated data.

We calculated the sensitivity/recall, specificity, and precision for each annotation level (Class, Mechanism, Group, and Model) at *E*-value thresholds in the range of [1e–50, 10] in base 10 increments. Next, we calculated the receiver operating characteristic (ROC) and Precision-Recall curves and quantified the area under the curve (AUC) for both ROC and Precision-Recall using linear interpolation between points. Hence, the sensitivity/recall, specificity, precision, and ROC-AUC and Precision-Recall AUC for all annotation levels are given in Table 1. The mean sensitivity and mean specificity were high across all levels of classification (between 97.5 and 100%) and thus, the resulting ROC-AUC was high (99.94 and 100%) for all classification levels. Therefore, Meta-MARC demonstrated both high sensitivity and specificity for the simulated data from leave-one-out cross-validation. The mean Precision-Recall AUC metric increased from the Model annotation level to the Class annotation level. The mean Precision-Recall AUC for the Model classification was 79.8%, for the Mechanism it was 87.2%, for the Group level it was 99.92% and for the Class level it was 99.97%. Supplementary Fig. 1 illustrates the increase in the mean Precision-Recall AUC over the different annotation levels. The Precision-Recall AUC improves in this manner due to the hierarchical nature of the annotation graph underlying the Meta-MARC model structure; as model annotation levels become less specific, the accuracy of the annotation improves. At the Model annotation level, the mean Precision-Recall AUC was 79.8%, whereas at the Class annotation level the mean Precision-Recall AUC was 99.97%, demonstrating a marked increase.

**Table 1:** Summary statistics as percentages for sensitivity, specificity, precision, recall, ROC-AUC, and PR-AUC for the predicted annotation

Annotation level	Performance metric	1st quartile	Median	Mean	3rd quartile
Class	Sensitivity/recall	99.01	99.50	99.31	99.96
	Specificity	99.95	99.98	99.96	100.00
	Precision	77.73	88.33	87.56	100.00
	ROC-AUC	99.94	99.98	99.97	100.00
	PR-AUC	80.32	92.86	88.95	100.00
Group	Sensitivity/recall	99.76	100.00	98.91	100.00
	Specificity	99.93	100.00	99.92	100.00
	Precision	74.74	100.00	85.65	100.00
	ROC-AUC	99.98	100.00	99.92	100.00
	PR-AUC	78.30	100.00	88.90	100.00
Mechanism	Sensitivity/recall	99.72	100.00	97.50	100.00
	Specificity	99.94	99.98	99.95	100.00
	Precision	73.79	88.17	85.58	100.00
	ROC-AUC	99.98	100.00	99.81	100.00
	PR-AUC	76.39	94.57	87.21	100.00
Model	Sensitivity/recall	99.72	100.00	97.50	100.00
	Specificity	97.43	99.93	100.00	100.00
	Precision	49.98	100.00	75.73	100.00
	ROC-AUC	99.97	100.00	99.91	100.00
	PR-AUC	52.78	100.00	79.80	100.00

All metrics excluding the AUC were calculated at the *E*-value threshold of 1e-25, which optimized the PR curve. We note that other thresholds might be useful depending on the false-positive tolerance of the use-case

### Description of functional metagenomic datasets

Functional metagenomics is an experimental design that allows for the characterization of metagenomic sequences that contribute to a given bacterial function^17,25–28^. In the case of AMR, this involves cloning fragments of metagenomic DNA into antibiotic-susceptible bacterial vectors that are grown on antibiotic-laden media. The bacteria that survive are then sequenced, resulting in a clonally amplified high-throughput sequence library containing one or more AMR genes.

We evaluated the performance of Meta-MARC on two functional metagenomic datasets, which we refer to as Soil and Pediatric, consisting of 169 samples and 219 samples with an average of 1.12 million and 1.98 million paired-end short reads, respectively (NCBI BioProject Accessions PRJNA215106 and PRJNA244044). The Soil and Pediatric datasets were generated by fragmenting metagenomic DNA into several-kb fragments, preparing fosmid libraries in *Escherichia coli* DH10B, growing these clones on various types of antibiotic-laden culture media at a concentration inhibitory to wild-type *Escherichia coli*, and sequencing any growing colonies (which are by definition phenotypically resistant to the antibiotic in the culture media)^29^. In this way, each sequenced colony represents phenotypically resistant bacteria, and the sequences within that fosmid necessarily contain one or more AMR genes (or other as-yet-unidentified sequences). As the antibiotic included in the culture media is known, each sequence from the surviving colony therefore has a resistance phenotype label; the classifier should, therefore, classify these sequences as AMR gene(s) known to confer phenotypic resistance to the specific class of antibiotics used in the corresponding culture media. However, because the original metagenomic fragments are longer than a single AMR gene, it can happen that a single fosmid contains multiple AMR genes; or alternatively, a phenotypically resistant fosmid may contain an AMR gene that has not yet been discovered, and thus is not included in the Meta-MARC model set. It follows that the phenotypic resistance profile for a functional metagenomics dataset is known, yet the specific AMR gene(s) from which each sequence read originated is not.

### Meta-MARC identifies more on-target sequences than competing methods

We compared the performance of the following methods using the Soil and Pediatric datasets: Meta-MARC on unassembled high-throughput sequence reads (Meta-MARC HTS Reads), Meta-MARC on assembled contigs (Meta-MARC Assembly), paired-end alignment to the MEGARes database using BWA^13^ (Alignment), and Resfams on assembled and translated contigs (Resfams). For Meta-MARC Assembly and Resfams, reads were assembled using IDBA-UD^30^. MetaGeneMark^31^ was used to translate the contigs into amino acid sequences using default parameters. The Meta-MARC *E*-value threshold for these experiments was set to 10, which was chosen based on the leave-one-out cross-validation results from the previous section. Since the phenotypic antimicrobial susceptibility information for the functional metagenomic samples is known (as discussed above), we refer to sequence reads as being classified on-target if the predicted AMR Class matched the known phenotypic resistance label; conversely, classifications are off-target if the predicted AMR Class did not match the phenotypic resistance label. We note that other standard performance metrics would be misleading because the sequence reads could have originated from the cloned *E. coli* genome, multiple AMR gene sequences on the same fragment, and/or a novel AMR gene sequence, thus obscuring whether off-target classification were true or false positives.

**Fig. 1:**
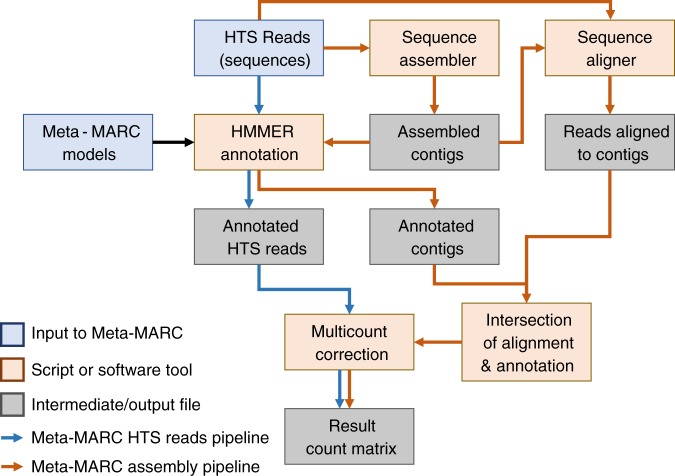
Comparison between the total number of classified reads (bar) and the number of on-target classified reads (crosshair) between the various methods. More specifically, Alignment (green), Meta-MARC HTS (orange), Meta-MARC Assembly (blue), and Resfams (purple) are compared for the Pediatric and Soil datasets. The *x*-axis is labeled by the confirmed AMR class. Of the 388 samples that comprise the Soil and Pediatric test sets, Alignment identified AMR targets in 161 samples (41.5%), Meta-MARC HTS identified 305 samples (78.7%), Meta-MARC Assembly identified 384 samples (98.9%), and Resfams identified 377 samples (97.2%)

**Fig. 2:**
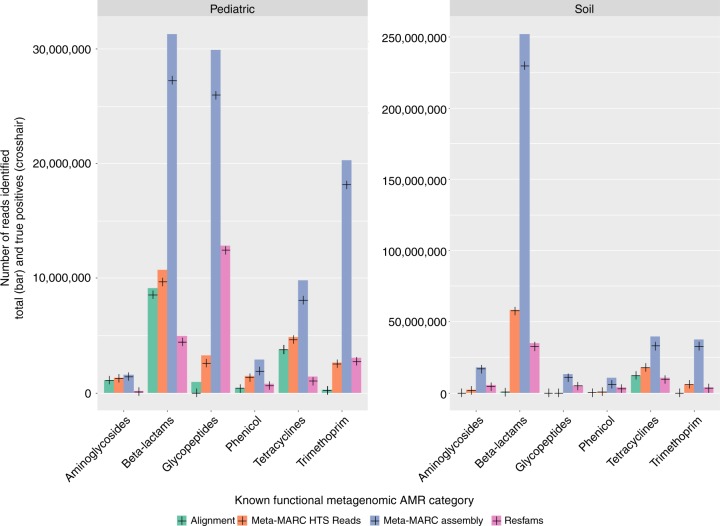
On-target classification rate of each method for each confirmed resistance class in the Pediatric and Soil datasets. Alignment and Meta-MARC HTS had improved performance on the Pediatric data than the Soil data, as evidenced by the higher quantiles of sample-wise classification rates shown here for the Pediatric data. Meta-MARC Assembly performed comparably to Resfams. Although alignment and Meta-MARC HTS classified fewer sequence reads overall, their on-target classification rates are comparable to Resfams and Meta-MARC Assembly on the Pediatric dataset

We illustrate the performance of Meta-MARC and competing methods on the Soil and Pediatric datasets as shown in Figs. 1 and 2, respectively. Meta-MARC Assembly had the highest on-target classification rate for both the Soil and Pediatric datasets (Fig. 1). Furthermore, the on-target classification rate of Meta-MARC high-throughput sequence reads was comparable to that of Resfams on assembled data, and higher than that of Alignment for every AMR Class. We note that both Alignment and Meta-MARC high-throughput sequence reads performed better on the Pediatric than the Soil datasets. Of the 388 samples that comprised the Soil and Pediatric test sets, Alignment identified AMR targets in 161 samples (41.5%), Meta-MARC high-throughput sequence reads305 samples (78.7%), Meta-MARC Assembly 384 samples (98.9%), and Resfams 377 samples (97.2%) (Table 2).

**Table 2:** True positive pairwise comparison for soil and pediatric data

		Compared to:			
		Alignment	Meta-MARC HTS Reads	Meta-MARC Assembly	Resfams
Method	Alignment	–	8.2%	0.8%	17.5%
	Meta-MARC HTS Reads	79.6%	–	1.3%	42.5%
	Meta-MARC Assembly	98.2%	97.7%	–	96.4%
	Resfams	81.4%	56.7%	3.1%	–

The table shows the percent of samples where a given method had a percentage of on-target classifications compared to another method. For example, Meta-MARC Assembly had a greater percentage of on-target classifications in 97.7% of the Soil and Pediatric samples compared to Meta-MARC high-throughput sequence reads

Pairwise comparison of each method’s performance on individual samples in the Soil and Pediatric datasets is presented in Tables 2 and 3. Table 2 shows the percentage of samples where one method had a higher percentage of on-target classifications in comparison to another method. Whereas, Table 3 shows the percentage of samples where one method had a greater number of on-target classifications in comparison to another—thus, the total number of classifications is not normalized by the total reads identified for Table 3. These results demonstrate that Meta-MARC Assembly classified a greater number of sequences correctly when compared to the competing methods. Meta-MARC had a higher on-target classification rate in comparison to Alignment in 98.2% of the samples, 97.7% in comparison to Meta-MARC high-throughput sequence reads, and 96.4% in comparison to Resfams (Table 2). Yet, it is worth noting that Resfams had a greater number of on-target classifications in 70% of the samples in comparison to Meta-MARC Assembly (Table 3). In comparison to the competing methods, Resfams and Meta-MARC Assembly performed comparably when the number of on-target classifications was considered. Lastly, we note that Alignment (which is arguably one of the most common forms of resistome classification) classified fewer sequence reads in each dataset but had high accuracy in those classifications, demonstrating high specificity due to its relatively low tolerance for sequence divergence from the reference. Furthermore, Alignment classified a negligible number of reads in the Soil dataset, showing poor performance on this specific dataset.

**Table 3:** Positive predictive value pairwise comparison for soil and pediatric data

		Compared to:			
		Alignment	Meta-MARC HTS Reads	Meta-MARC Assembly	Resfams
Method	Alignment	–	31.4%	33.8%	30.4%
	Meta-MARC HTS Reads	55.7%	–	64.7%	44.6%
	Meta-MARC Assembly	65.2%	33.8%	–	29.1%
	Resfams	68.3%	50.0%	70.1%	–

The table shows the percent of samples where a given method had a higher number of on-target classifications (regardless of total number classified) compared to another method. For example, Meta-MARC Assembly had a greater number of on-target classifications in 33.8% of the Soil and Pediatric samples compared to Meta-MARC high-throughput sequence reads

### Description of shotgun metagenomics data

To assess the performance of Meta-MARC on standard (i.e., non-functional) metagenomic data, we utilized a dataset consisting of 87 samples that were collected from various phases and various sample matrices throughout beef cattle production (NCBI BioProject Accession PRJNA292471)^32^. The dataset was chosen due to the diversity of sample types and the fact that the samples originated from environments and host populations that have not been extensively studied. The data therefore likely contain a greater proportion of sequences that are divergent from those used to train the Meta-MARC HMMs. The 87 samples in this dataset were processed for total DNA extraction and sequenced on an Illumina HiSeq, resulting in 407.7 Gb of sequence data (average 46.3 M reads per sample, range 12.0–93.4 M.

### Meta-MARC tolerates more genetic variation in shotgun metagenomic data

**Fig. 3:**
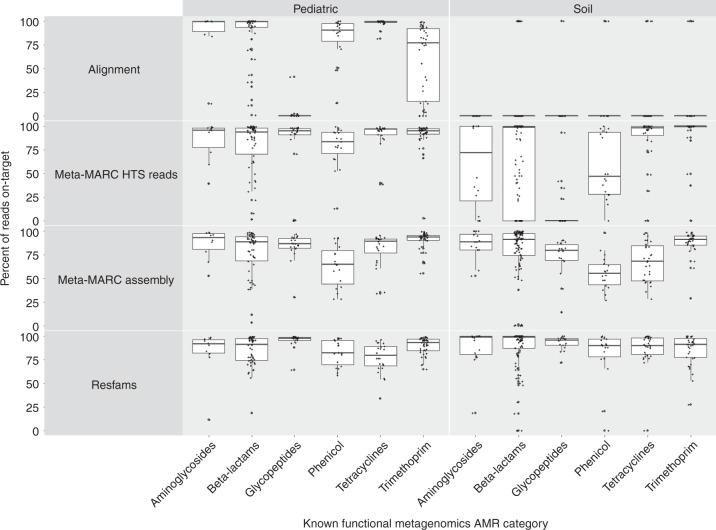
AMR class abundance for the shotgun metagenomic dataset of Noyes et al.^32^. Alignment (green), Meta-MARC HTS (orange), Meta-MARC Assembly (blue) and Resfams (purple) are compared. Meta-MARC Assembly identified the greatest number of reads in 11 out of 13 resistance classes. For the aminoglycosides and multi-drug resistance genes, Resfams identified a greater number of reads with Meta-MARC containing the second largest number of identifications in those classes. Overall, Meta-MARC Assembly detected 42-fold more reads than Alignment, and 1.5-fold more reads than Resfams

Each of the 87 samples in the shotgun metagenomic dataset were analyzed by each comparative classification method, and the number of sequence reads classified within each Class is presented in Fig. 3. Meta-MARC Assembly achieved a 42-fold increase in total number of sequences classified compared to Alignment and a 1.5-fold increase compared to Resfams across all 87 samples. Whereas Alignment and Resfams primarily classified sequences into the tetracycline and macrolide, lincosamide, and streptogramin (MLS) Classes, both Meta-MARC high-throughput sequence reads and Meta-MARC Assembly were able to identify sequences from a diverse and variable number of resistance Classes. In particular, Meta-MARC classified a substantial number of reads into six resistance Classes that Alignment and Resfams did not. Furthermore, Meta-MARC Assembly classified the greatest number of reads in 11 out of 13 resistance Classes.

In addition to comparing the number of reads that aligned to each resistance Class, we assessed the amount of genetic variation that each method tolerated in its classification. To perform this assessment, we first calculated the number of single nucleotides that differed between each sequence read, and the respective contig (Meta-MARC Assembly and Resfams), model (Meta-MARC high-throughput sequence reads) or reference sequence (Alignment). For Alignment, this calculation was accomplished by counting all mismatches identified in the CIGAR field of the sequence alignment map (SAM) file obtained from BWA^13^. For Meta-MARC high-throughput sequence reads, this calculation was accomplished by identifying the sequence read alignment start and end positions in the respective model consensus string (i.e., the consensus of the alignment profile used to create the model), comparing the read to the model consensus string at each nucleotide position within this region, and counting the number of nucleotides that mismatched. We note that the start and end positions of the alignment to the consensus string is given as output from HMMER. This calculation was performed similarly for Meta-MARC Assembly and Resfams, with the main difference being that the alignment between the sequence read and the consensus string was not given as output from HMMER because the contigs—and not the individual reads—had been classified. Therefore, the start and end position of each read to the consensus string had to be inferred via alignment to the contig. Thus, for Meta-MARC Assembly and Resfams, the calculation was accomplished by aligning the sequence reads to the assembled contigs using the Burrows Wheeler Aligner^13^ in order to determine the position in the contig where each read aligned. This information was then combined with the start and end position of the contig with respect to the model consensus string to obtain the start and end positions of each read within the consensus string. Finally, each nucleotide position within this region was compared and the number of mismatches was obtained. After counting the total number of mismatches for each model, we determined the mean for each method, and analyzed these means using a linear mixed effects regression model with the following independent covariables: the method type as a categorical fixed effect (with alignment as a reference value), and class annotation level as a random effect (varying intercept). The same data were also analyzed using a two-sided Wilcoxon rank sum test and corrected for multiple test correction using the Bonferroni method^33^.

**Fig. 4:**
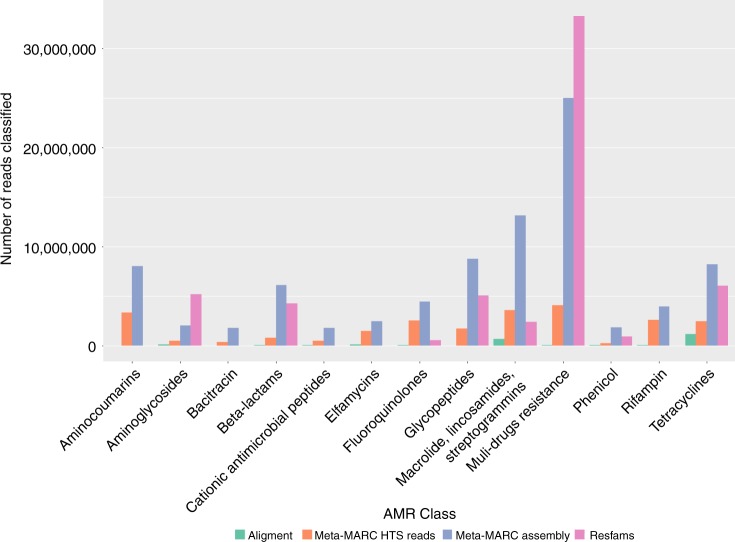
Comparison between the average number of variations between a read and consensus sequence (contig or reference). Counts of the non-major allele were determined for each genomic position by method and summed by AMR class category and sample. The median value for the methods utilizing assembly and alignment was ~7 variations per read on average. We note that the assembly methods also utilized alignment to map the DNA sequence reads back to the consensus contigs, which contributed to the reduction in allowed variation. The median value for Meta-MARC HTS was threefold higher than competing methods. Meta-MARC HTS was significantly different (***P < 0.001) compared to every other method via a Wilcoxon rank-sum test, corrected for multiple testing by the Bonferroni method. The Wilcoxon rank-sum test was performed using 1891 independent Class-level node and sample combinations per group tested

We determined that Alignment, Meta-MARC Assembly, and Resfams all exhibited sample-wise median genetic variation allowance between 5 and 7 nucleotides per read (Fig. 4), with Meta-MARC Assembly and Resfams being the least tolerant of genetic variation. This is unsurprising, as both of these methods rely on short sequence alignment to calculate the genetic variation, and both require assembly. Since the goal of genome assembly is to construct a single consensus sequence from high-throughput sequence reads as a representation of the genome, genetic variation will not be represented in the assembled contigs. The difference in sequence divergence tolerance between Alignment and Meta-MARC high-throughput sequence reads was calculated as a coefficient of 22.42 (0.72 Std. err.), controlling for the resistance Class as a random effect. This can be interpreted as an average increase of 22 mismatches per read using Meta-MARC high-throughput sequence reads over Alignment, which is a threefold increase. Hence, Meta-MARC high-throughput sequence reads allowed for significantly more genetic variation than any other method as assessed by the Wilcoxon rank sum test (adjusted *P* < 0.001 for all pairwise comparisons).

### Meta-MARC recovers highest percentage of sequences in divergent simulated data

The classification of a false positive is nontrivial in this context since the goal of Meta-MARC is to identify genes that are divergent from MEGARes (or any other database). Thus, in this section, we created in silico genes that have increasing divergence from the genes in MEGARes and simulated sequence reads from these and other additional prokaryote genomes. The addition of other prokaryote genomes allows for background noise that is common in shotgun metagenomics experiments. Specifically, we did the following: (1) we randomly selected gene X from the MEGARes database, (2) we replaced a fraction (equal to [Y × length of gene X]) of nucleotides for gene X both contiguously (at a random location) and noncontiguously at random without replacement, where the insertion or random mutation was generated from selecting A, C, G, T with equal probability, (3) we made *Z* copies of the modified gene, (4) we added these copies to six genomes of *Escherichia coli* (Accession numbers: AP009048, CP009789, CP010441, CP010445, U00096), and lastly, (5) we simulated sequence reads from these sequences using ART with parameters as previously described. We assessed all methods using various values of X, Y, and Z, i.e., we repeated this generation for *Y* = 0.1, 0.25, 0.5, 0.7, and *Z* = 5, 25, 50 and 70 different genes (of varying sizes). In total, there were 70 × 24 = 1680 experiments. Lastly, we ran all four methods for each experiment. For each method, we determined whether the gene was identified correctly at the Class, Mechanism, Group, and Model levels. All data and source code for these experiments are available at the publication GitHub repository.

**Fig. 5:**
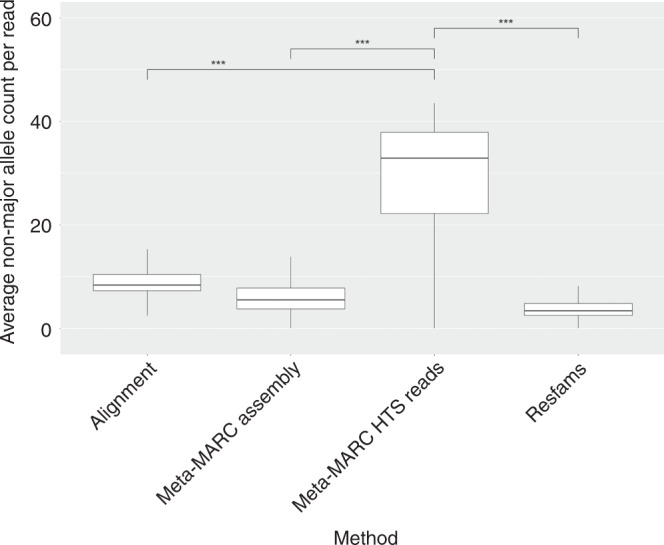
Meta-MARC recovers > 90% of simulated reads despite many random point mutations and moderate (25%) contiguous randomization. Percent of genes classified correctly (recovered) is negatively associated with the proportion of the reference sequence randomized before simulation for all methods. Contiguous randomizations (insertion/deletions) demonstrate a consistent negative trend for all methods. Noncontiguous, random point mutations are tolerated well by Meta-MARC Assembly, moderately well for Meta-MARC HTS, and poorly for Alignment and Resfams. Each data point represents a unique annotation Class. The lines were fit using LOESS regression in R, and the gray area is the standard error of the LOESS fit

The results show that Meta-MARC Assembly recovers the highest percentage of simulated sequences even when the target sequences had high mutation rates (Fig. 5). For the contiguous mutations, all four methods demonstrated a negative linear trend, decreasing in their ability to recover simulated sequences with increasing length of the mutated region. For the noncontiguous random mutations, Meta-MARC Assembly performed with > 90% average recovery rate at all mutation levels examined. Meta-MARC high-throughput sequence reads performed with > 90% average recovery rate as high as 50% mutation level in the target sequences, however, its performance dropped substantially at the 70% mutation level. Alignment and Resfams had quickly declining recovery rates as mutation level increased. Recovery rates were comparable across annotation levels (Class, Mechanism, Group, and Model) (see Supplementary Figs. 2–4). Gene copy number did not affect recovery rate.

### Comparison of CPU-time and memory usage

In this section, we evaluate Meta-MARC’s ability to scale with input size. To accomplish this evaluation, sequence data were first filtered to exclude host (i.e., *Bos taurus*) DNA; next, we selected five test datasets with varying numbers of sequence reads. All experiments in this section were performed on a server running Debian Linux 3.16 with Intel(R) Xeon(R) CPU E5–2680 v2 @ 2.80 GHz with 324 GB RAM. The parallelized portions of each algorithm were run with 40 threads.

**Fig. 6:**
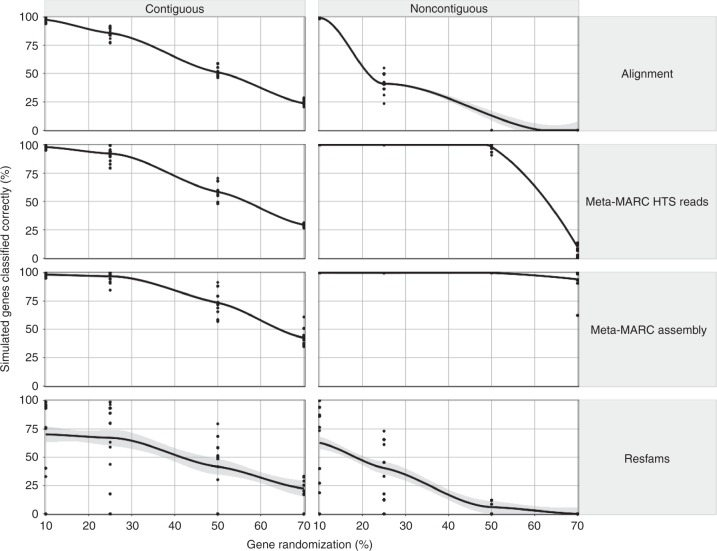
The runtime required by each algorithm as the size of the input increases, as described in the text

In Fig. 6, we list the wall time versus data input size for each method. It is important to note that the wall times for Resfams and Meta-MARC Assembly do not include the time required for assembly. As expected, Meta-MARC high-throughput sequence reads was the slowest algorithm; it required up to 28 h on the largest dataset, in comparison with Alignment, which required less than an hour. Thus, the increased sensitivity of Meta-MARC high-throughput sequence reads over its competitors comes with a computational cost.

In Supplementary Fig. 5, the peak memory of each algorithm is shown versus the size of the input. Meta-MARC high-throughput sequence reads uses more memory than its competitors; although the memory requirements of assembly are not shown. Meta-MARC high-throughput sequence reads uses up to 250 times more memory than Alignment. The largest dataset was size 13 GB on disk; for this input, Meta-MARC high-throughput sequence reads required less than 24 GB of memory, which is within a factor of 2 of the disk size of its input.

## Discussion

Resistome classification from metagenomic data is increasingly utilized to better understand AMR ecological dynamics and to track AMR dissemination and evolution across time and space. Given the importance of this task, the research community urgently needs more accurate tools to detect and classify short-read sequences originating from AMR genes. By combining machine learning classification with a well-annotated and structured database, we have demonstrated that a high level of AMR classification sensitivity and specificity can be achieved. Using simulated data for leave-one-out cross-validation, we showed that our method (named Meta-MARC) reached mean sensitivity and specificity values >97% for all levels of the annotation. We emphasize that the focus of this work is on the sensitivity and precision of the methods assessed, since these are the more difficult metrics to optimize in a classification problem with many classes. Specificity in such problems will be high by random chance, however detecting (sensitivity) and correctly classifying (precision) sequences to one of many categories is much more difficult.

In addition, Meta-MARC achieved a higher on-target classification rate than comparable, existing methods when used to classify AMR sequences obtained from two functional metagenomic studies. Of particular note was the difference in on-target classification rate among the various methods when comparing results for the Pediatric and Soil datasets. While the Pediatric dataset enjoyed a higher on-target classification rate than the Soil data across all methods, the largest discrepancy was witnessed between Alignment and the two assembly-based methods (i.e., Resfams and Meta-MARC Assembly). When analyzing the Pediatric dataset, the Alignment method classified a comparable number of reads to the other methods (Table 2). However, Alignment was ineffective for the majority of samples in the Soil dataset. We hypothesize that the reason for this discrepancy could be that the pediatric resistome is better represented in current AMR databases because of study bias in the literature, and thus AMR genes in the Pediatric samples were more homologous with database sequences in MEGARes. In contrast, the Soil samples likely contained a higher diversity of less well-characterized AMR genes^17,24,25^. As the Alignment method requires a near-exact match between sequence read and reference, this method therefore performed much better on the Pediatric than the Soil samples, whereas the more probabilistic methods were able to tolerate such divergence and thus performed less discrepantly on the Pediatric and Soil datasets.

To further assess the classification capability of Meta-MARC for sequences with high divergence from reference databases, we evaluated the performance of Meta-MARC and competing methods on shotgun metagenomics data collected and sequenced from beef production facilities. Since this environment has not been a frequent source of AMR sequences deposited in reference databases, we expected this data to be divergent from the currently available AMR databases. In our experiments, Alignment classified far fewer reads than all other methods and in certain instances this difference was dramatic, e.g., Meta-MARC Assembly classifying 42-fold more reads than Alignment (Fig. 3). Moreover, for most Classes of AMR, Meta-MARC Assembly identified more putative AMR sequences than did Resfams. This latter point illustrates the performance gained by using a well-structured DNA AMR database, as the only major differences between the Resfams and Meta-MARC Assembly approaches were the database used to construct their respective HMM models and the DNA versus amino acid level classification.

Meta-MARC Assembly, Resfams, and Alignment relied on a short-read aligner for either read classification or read alignment to the models or contigs. This resulted in a significant reduction in the amount of genetic variation identified in sequence reads compared to Meta-MARC high-throughput sequence reads, the latter of which was able to identify threefold higher genetic variation (Fig. 4). However, the total number of reads identified by Meta-MARC high-throughput sequence reads was frequently lower than Meta-MARC Assembly and Resfams. Meta-MARC Assembly had the highest sensitivity of the four methods assessed here, whereas Alignment was the least performant but had high specificity with regard to reads identified. Meta-MARC’s high-throughput sequence reads classification rate was intermediate between Alignment, Resfams, and Meta-MARC Assembly, but it identified significantly more genetic variation than the other methods. These findings are relevant for metagenomic investigations of AMR ecology because the use of classification methods with low sensitivity could result in a biased understanding of the important dynamics regarding the control of AMR in populations—particularly in environments where selective pressure for genetic divergence from known AMR sequences is high. Future investigations utilizing a resistome approach should weigh the benefits and limitations of each of these methods with respect to the specific research questions being asked.

The Meta-MARC Assembly and high-throughput sequence reads methods were shown to be robust to sequence divergence away from the reference database (Fig. 5). In particular, these methods were able to recover mutated target sequences containing many single-nucleotide polymorphisms that occurred noncontiguously at random throughout the target sequence. We observed that the ability of alignments to recover these same mutated sequences quickly decreased as mutation rate increased, further supporting the view that Alignment is a specific method but lacking in sensitivity in the face of divergence away from reference. Resfams showed poor recovery rates in the face of random mutations. We hypothesize that this is due to the introduction of premature stop codons and substantial amino acid changes that single nucleotide polymorphisms can cause. DNA-based classifiers like Meta-MARC and Alignment appear to be more robust to noncontiguous random mutation. However, all methods were impacted negatively by mutation of *contiguous* regions of the target sequence. This is expected, since HMMs detect target sequences by finding contiguous regions of high similarity/probability; thus, when large contiguous regions are mutated, the algorithm does not perform well.

We note that the increased sensitivity gained by running the Meta-MARC HMMs with a sensitive *E*-value threshold (e.g., *E*-value 10) may also lead to an increased number of false-positive classifications. This limitation of Meta-MARC, and machine learning classifiers in general, necessitates careful consideration of the read classifications by Meta-MARC. It would be prudent to consider additional methods to confirm that the reads identified by Meta-MARC are, in fact, related to AMR genes. Yet, conditions that lead to either shallow sequencing coverage or divergent sequences frequently necessitate the use of a more sensitive classification method. For example, several water samples from the Noyes et al. dataset used in this study^31^ contained relatively small numbers of reads (<13 M) and thus may require a more sensitive classification method than alignment. In this analysis, we have demonstrated that the method used for AMR classification from metagenomic sequence data has a substantial impact on the number of predictions made, with short-read alignment-based methods consistently exhibiting the lowest classification rates and the lowest tolerance for genetic variation. Conversely, probabilistic machine learning methods consistently identify more AMR-originating sequence reads while tolerating a higher level of sequence divergence. These differences are particularly stark when the sequence data originate from environments not commonly represented in current AMR databases and/or from highly divergent sequences compared to those already reported. These findings mirror widely acknowledged low classification rates from high-throughput sequence data with respect to microbiome sequencing^15,26,27^.

While classification will likely be improved with the development of more comprehensive AMR databases, immediate improvement in AMR identification rate can be made through the use of probabilistic classifiers, as demonstrated here with Meta-MARC. Lastly, we note that although the methods here were compared separately on the same datasets, it would be possible to combine some of the methods into an integrated pipeline for AMR classification. One possible combination is to first align all sequence reads to an AMR database using a short-read aligner (high specificity), and then to classify remaining unaligned reads using Meta-MARC (high sensitivity). Combinations such as these warrant further investigation and should be considered as viable options for future resistome research. Ultimately, however, choice of classification method should be driven by study objectives, sampling environment (i.e., well-studied or novel), and the appropriate trade-off of sensitivity, specificity, on-target classification and computational resources.

## Methods

### Overview of Meta-MARC

**Fig. 7:**
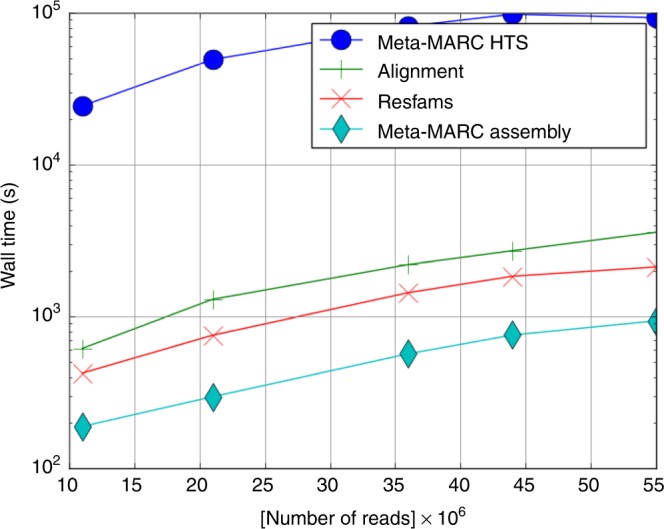
Meta-MARC utilizes two workflows to classify and count HTS data: Meta-MARC HTS Reads and Meta-MARC Assembly. Meta-MARC HTS Reads Pipeline: HTS reads are input as FASTQ files to be classified by the HMMER software against the pre-built Meta-MARC Models. Resulting counts are processed to correct for multiple classifications; for example, if a single input read is classified to multiple models, the count for that read is divided evenly between the models to maintain a 1:1 input to output ratio. Meta-MARC Assembly Pipeline: HTS reads are de novo assembled to produce contigs. The HTS reads are then aligned back to these assembled contigs to produce an alignment file. The assembled contigs are annotated by HMMER against the Meta-MARC Models. Using the alignment information, HTS reads that also overlap a Meta-MARC model annotation in the assembled contigs are counted. The resulting counts are processed to correct for multiple classifications as described above. The final output of both pipelines is a corrected count file, listing the number of HTS reads classified to each Meta-MARC Model

Meta-MARC accepts as input one or more sequences and predicts the AMR sequence of origin. The input sequences can be high-throughput sequence reads from shotgun metagenomics, functional metagenomics, whole-genome sequencing, or assembled contigs. Meta-MARC was constructed using the MEGARes database, which is a DNA database of AMR genes that was manually curated from existing AMR databases and annotated for the purpose of creating machine learning classifiers, including use of an acyclic ontological structure^12^. Meta-MARC itself is a group of HMMs, where each HMM corresponds to a set of AMR genes clustered by sequence similarity and annotated using MEGARes. Thus, the models are constructed by clustering the genes in MEGARes based on sequence similarity and training an HMM for each cluster. This produces a set of machine-learning models that together classify sequence data by AMR model. Figure 7 gives a schematic view of Meta-MARC.

Meta-MARC maintains the annotation structure found in MEGARes, allowing for classification of sequences at the AMR Class, Mechanism, and Group levels. The graph underlying the annotation of each gene in MEGARes is a tree structure with the following four levels (starting from the highest level): AMR Class, Mechanism, Group, and sequence. Each level refers to a specific pharmaceutical or biological classification group. For example, a *bla*-SHV gene might be classified as: beta-lactams (Class level), Class A beta-lactamase (Mechanism level), SHV (Group level), and the specific gene sequence (sequence level). The tree structure of the annotation graph implies that no cycles exist in the underlying graph, meaning that no parent nodes share the same child node. For example, the sequences associated with Class A beta-lactamase Mechanisms can belong to two different beta-lactamase Groups, but a single *bla*-SHV gene cannot belong to two (or more) Class A beta-lactamase Mechanisms. The implication of this structure is that the corresponding machine-learning method classification can be accomplished by making a prediction at the lowest level (AMR sequence) and then aggregating up the graph without correcting for dependencies and without ambiguities in determining the higher level annotations.

Ideally, clustering based on sequence similarity would result in self-consistent clusters, i.e., groups of sequences that belong to the same AMR Class, Mechanism and Group. While most of the Meta-MARC models are independent in this manner, several models contain sequences from multiple groups and mechanisms. This is a result of a necessary compromise between AMR sequence similarity and the current classification scheme utilized by biologists engaged in AMR research. For example, some sequences are currently annotated by biological classification schema as *bla*-SHV and *bla*-TEM beta-lactamase genes but contain >80% nucleotide sequence identity; such sequences have been included in the same model to reduce classifier confusion and false-positive classification rate. At the Class level, however, all clusters were self-consistent, i.e., contained only sequences annotated within the same Class. As a result, to avoid dependence between model classifications, all analyses described here were evaluated at the class annotation level, but results for the lower levels were made available as well for completeness.

### Construction of the Meta-MARC models

Sequences from the MEGARes database v1.00 in August 2016 were used as the foundation for the construction of the Meta-MARC models. Prior to multiple-pairwise sequence alignment using USEARCH^34^, the sequences were separated into three categories based on biological relevance and bioinformatics requirements*:*

### Group I (284 models, 2905 MEGARes sequences)

Group I models correspond to clusters of sequences that contain more than two unique sequences belonging to a single type of antimicrobial resistance (and therefore Group I models exclude multi-drug resistance genes). Group I models represent the ideal scenario for machine-learning classification, because robust HMM construction necessitates more than two sequences, and each of the sequences belong to the same AMR Class. Therefore, Group I are the high-confidence models in Meta-MARC.

### Group II (108 models, 307 MEGARes sequences)

Group II models contain sequences that require single- or multi-locus mutational verification to confirm the presence of potential AMR properties. Like Group I, this group of models excludes multi-drug resistance genes. These sequences are typically allelic variations of so-called “housekeeping” genes, such as the beta-subunit of the bacterial ribosome (*rpoB*) or a subunit of DNA topoisomerase IV (*parC*). Since these genes are commonly found in non-resistant strains of bacteria, it would be inappropriate to include them in the resistome without further identification of specific single-nucleotide polymorphisms (SNPs) and/or mutation(s) associated with a resistant phenotype. The literature regarding such SNPs/variants is of variable quality and quantity, and there currently exists no centralized, validated catalog of all potential resistance-conferring SNPs for these house-keeping genes; this makes it impossible to perform robust SNP-level verification. Therefore, all sequences identified by the Group II models were excluded from our analysis and results. However, we include this group of models so that Meta-MARC users have the option to detect these genes and perform their own post-processing mutational verification.

### Group III (675 models, 1073 MEGARes, 28,603 BLAST sequences)

The third group of models includes sequences belonging to the multi-drug resistance genes, such as resistant porin proteins or Major Facilitator Superfamily (MFS) efflux pumps. Additionally, all models that contained only a single sequence after clustering were included in this group. Since robust HMM classifiers cannot be developed based on a single representative sequence, these singleton sequences were augmented by performing a nucleotide BLAST against the non-redundant nucleotide database. Sequences from BLAST results that fell between 95 and 99% identity to the query (singleton) sequence at the nucleotide level were then included in the clustering and model construction process for the Group III models. Each sequence added by BLAST search to these models was labeled as “blast_augmented” in its FASTA header. Group III models, therefore, represent resistance genes that could cause resistance but likely have other purposes in the target organisms, such as efflux of other, non-antimicrobial molecules.

After sequences were separated into these groups, and each group was clustered at 80% nucleotide identity using USEARCH^34^ with the command-line flags *cluster-fast*, *id 0.8*, *sort length*, *msaout*, and *uc*. Eighty percent nucleotide identity was selected based on a non-comprehensive analysis of how well the sequences clustered while still maintaining the integrity of the MEGARes annotation graph. Next, the multiple-pairwise alignments resulting from USEARCH clustering were used as input to the HMMER *hmmbuild* command. The individual HMM files were then combined into the Groups I, II, and III model files using the HMMER *hmmpress* command. The source code for performing the model building, as well as all sequence and intermediate files for the model construction process, can be found on the build branch of the Meta-MARC source code repository. Production HMM files can be found in the master branch of the Meta-MARC repository.

### Reporting summary

Further information on research design is available in the Nature Research Reporting Summary linked to this article.

## Supplementary information

Supplementary Material

Reporting Summary

## Data Availability

The datasets used in this study are publicly available at the National Center for Biotechnology Information BioProject Accessions PRJNA215106, PRJNA244044, and PRJNA2924710.

## Code availability

Code involved in the construction and running of the Meta-MARC hidden Markov models is publicly available on GitHub (https://github.com/lakinsm/meta-marc)^35^. Code involved in data analysis and preparation of the manuscript is also publicly available on GitHub (https://github.com/lakinsm/meta-marc-publication)^36^.
