# Supplementary material for: Hierarchical Hidden Markov models enable accurate and diverse detection of antimicrobial resistance sequences
Source: Commun Biol. 2019 Aug 6;2:294. doi: 10.1038/s42003-019-0545-9 (PMC6684577; doi:10.1038/s42003-019-0545-9)
Supplement: Supplementary file 1 — Supplementary Material [file 42003_2019_545_MOESM1_ESM.docx]

**Supplementary Figures**


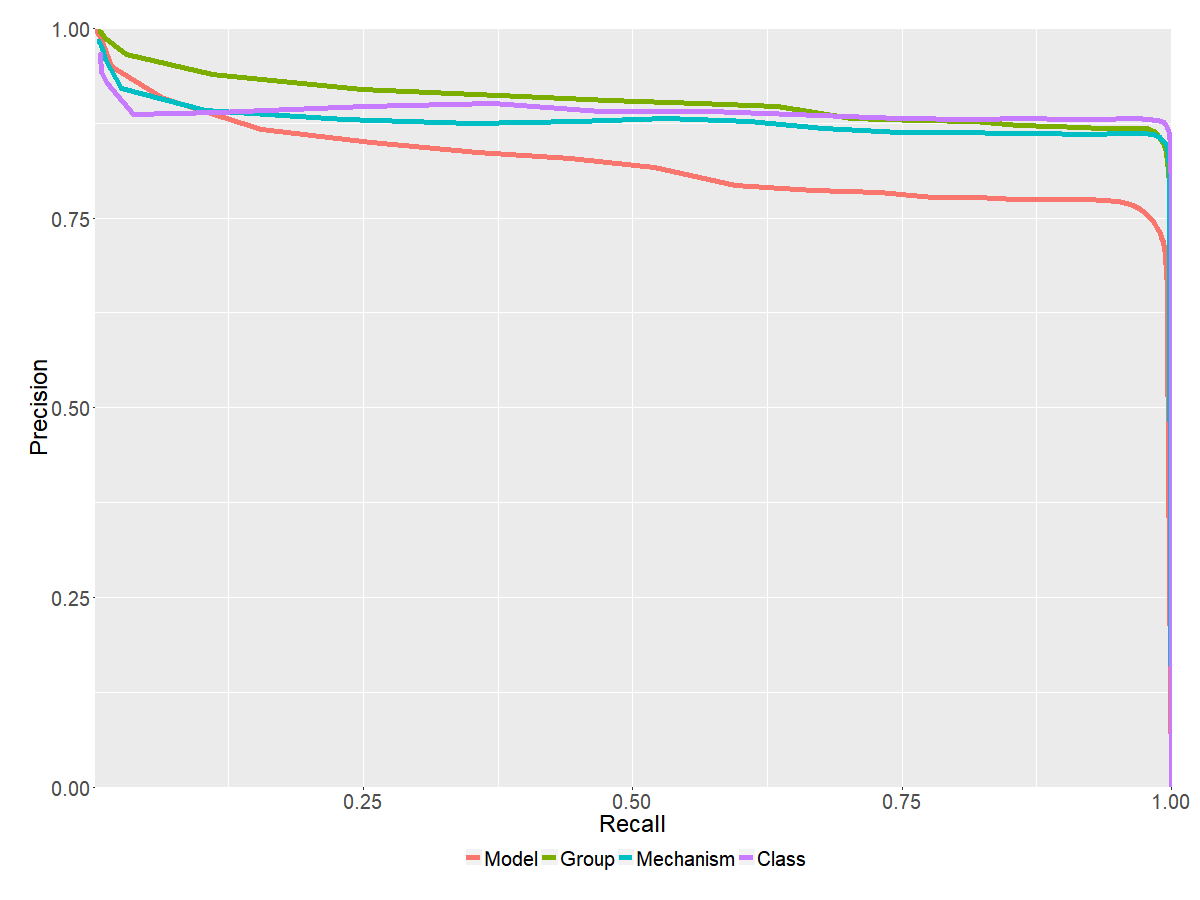


Supplementary Figure 1. PR curves for each Meta-MARC hierarchical annotation level: Model (red), Group (green), Mechanism (blue), and Class (purple). Classification performance on average improves with classification to the higher levels of the hierarchical annotations when evaluated at reasonable E-value thresholds (approximately [1e-25, 10]).


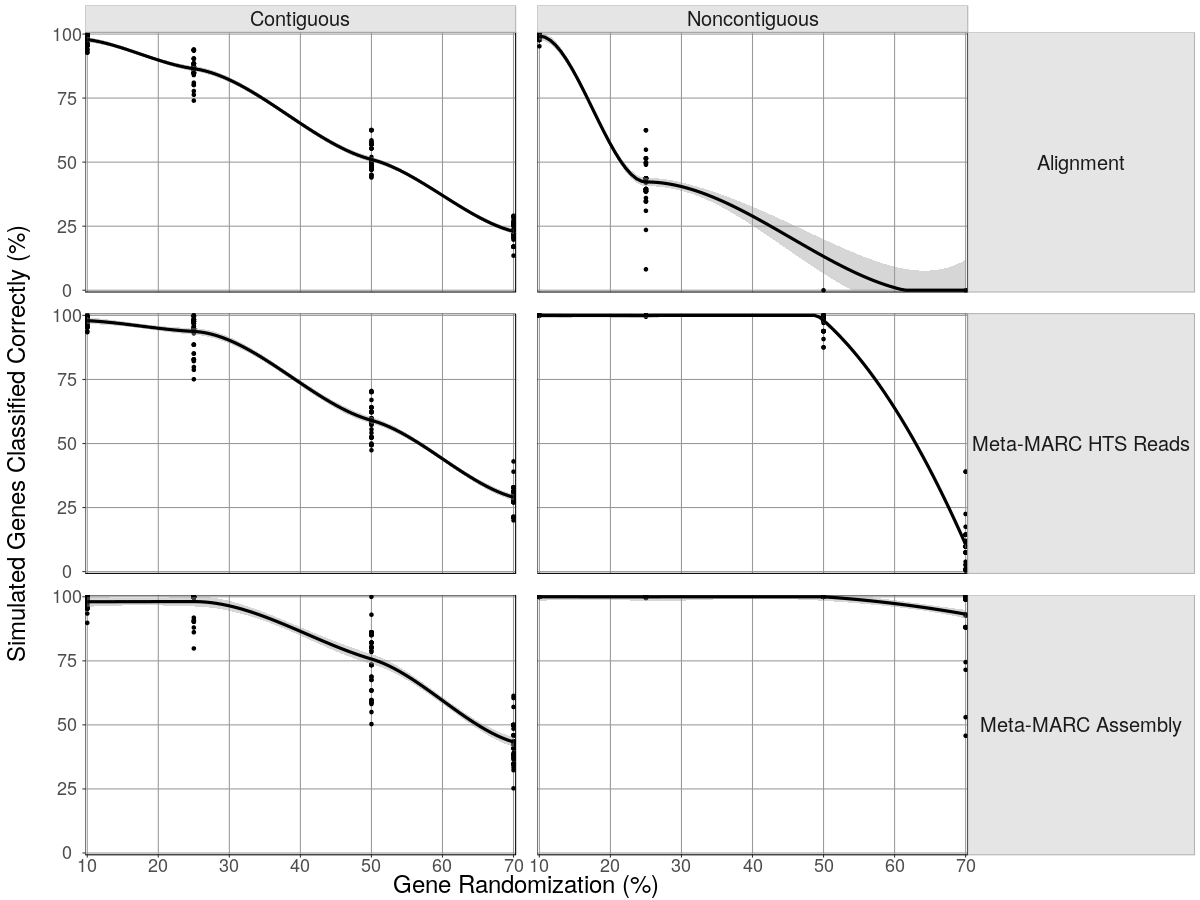


Supplementary Figure 2. Mechanism-level results for the for the simulated mutation experiment in section 3.4. Results follow the same trends as in Figure 7, however the standard error increases with decreasing annotation level.


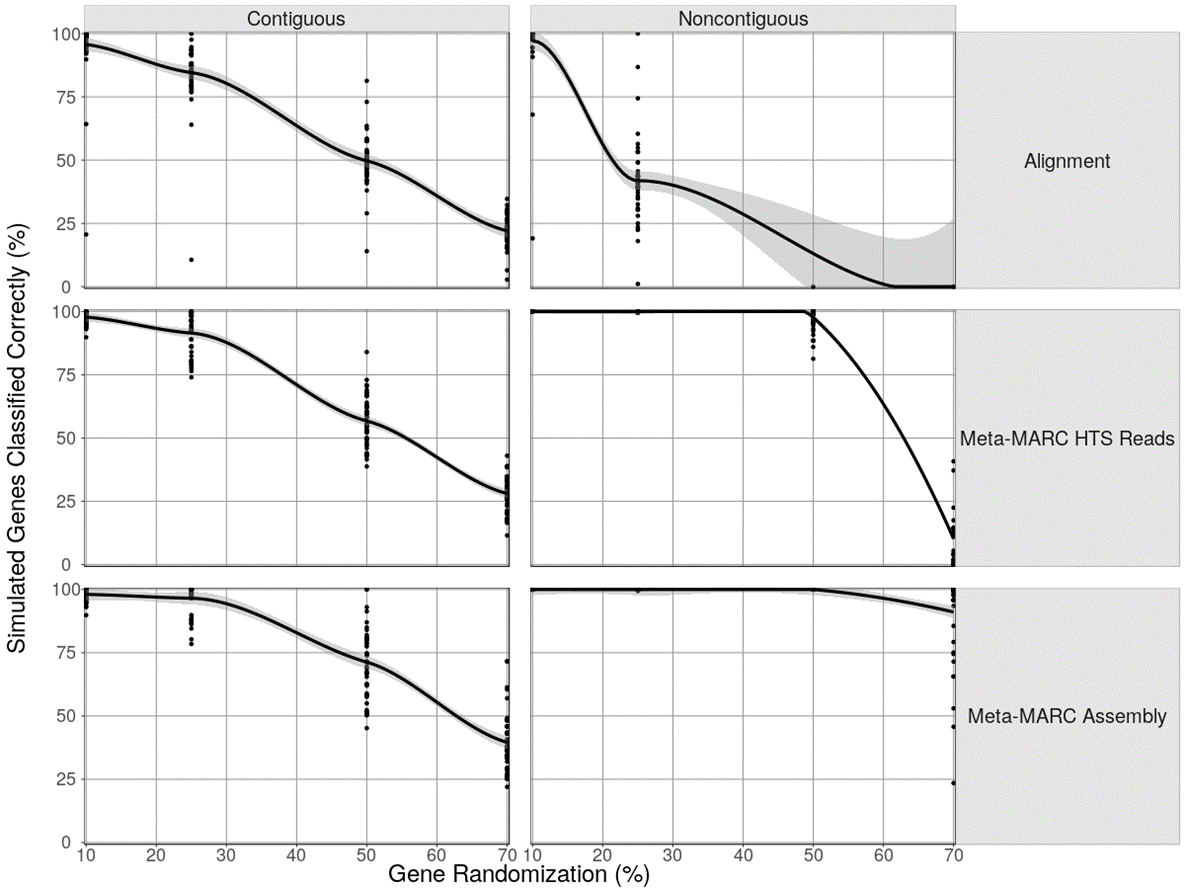


Supplementary Figure 3. Group-level results for the for the simulated mutation experiment in section 3.4. Results follow the same trends as in Figure 7, however the standard error increases with decreasing annotation level.


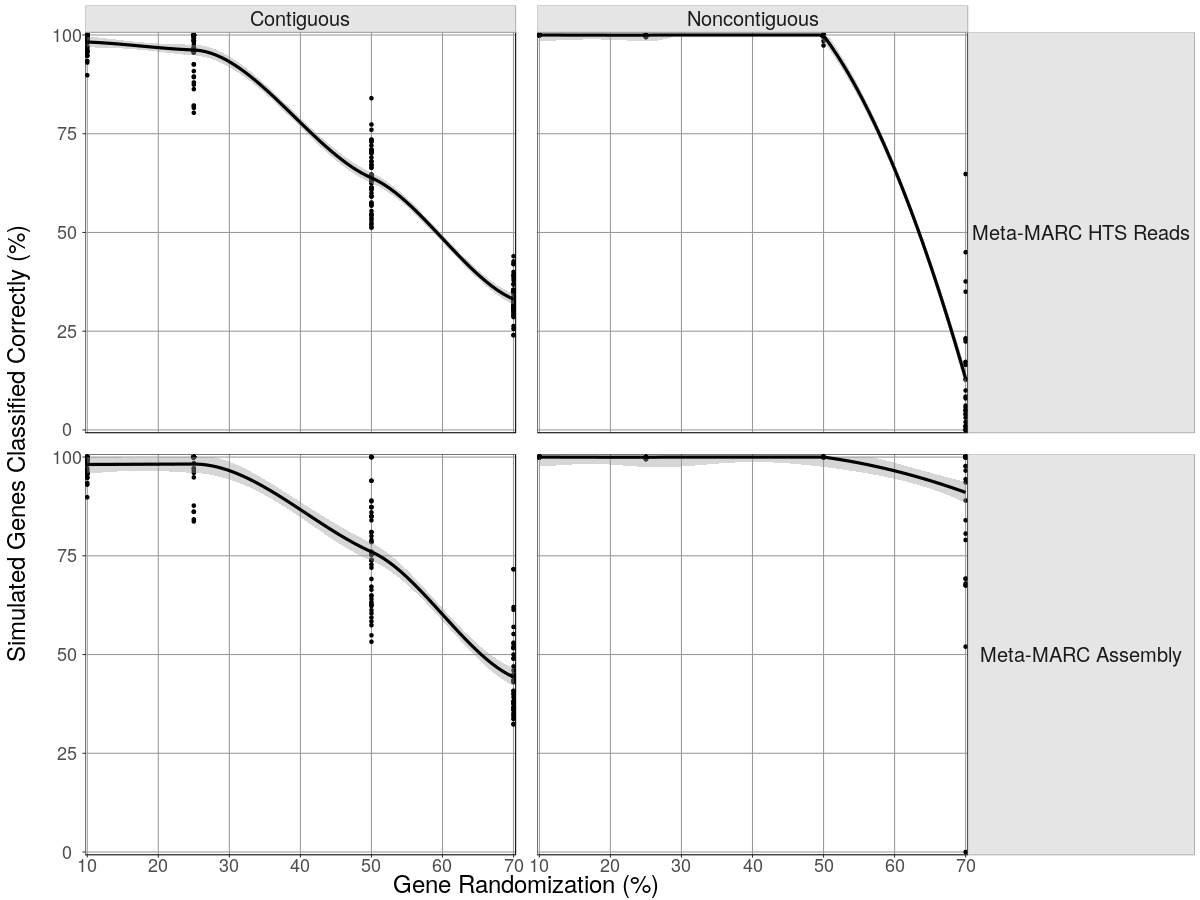


Supplementary Figure 4. Model-level results for the for the simulated mutation experiment in section 3.4. Results follow the same trends as in Figure 7, however the standard error increases with decreasing annotation level.


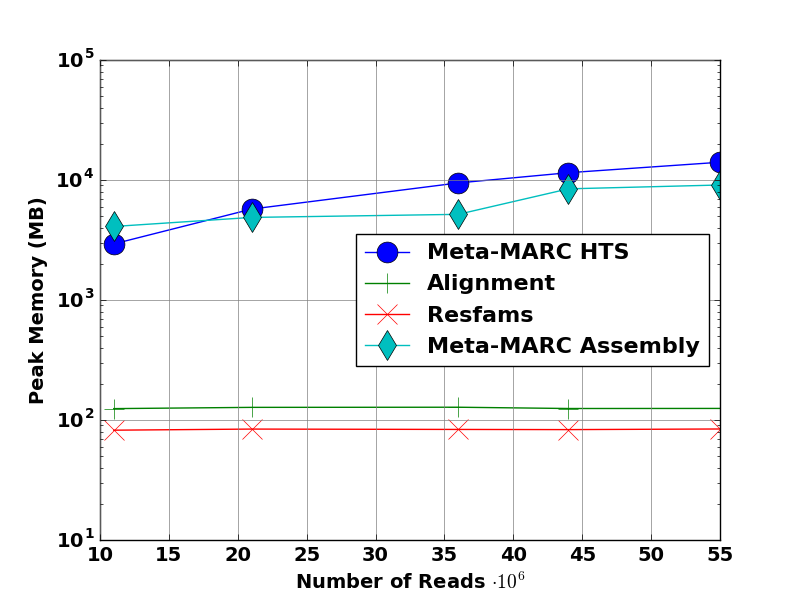


Supplementary Figure 5. The peak memory required by each algorithm as the size of the input increases, as described in the text.
