# Supplementary material for: Hierarchical Hidden Markov models enable accurate and diverse detection of antimicrobial resistance sequences
Source: Commun Biol. 2019 Aug 6;2:294. doi: 10.1038/s42003-019-0545-9 (PMC6684577; doi:10.1038/s42003-019-0545-9)
Supplement: Supplementary file 2 — Reporting Summary [file 42003_2019_545_MOESM2_ESM.pdf]

natureresearch

Corresponding author(s): Christina Boucher

Last updated by author(s): Jun 25, 2019

Reporting Summary

Nature Research wishes to improve the reproducibility of the work that we publish. This form provides structure for consistency and transparency in reporting. For further information on Nature Research policies, see Authors & Referees and the Editorial Policy Checklist.

Statistics

For all statistical analyses, confirm that the following items are present in the figure legend, table legend, main text, or Methods section.

n/a	Confirmed	
☐	☒	The exact sample size (*n*) for each experimental group/condition, given as a discrete number and unit of measurement
☐	☒	A statement on whether measurements were taken from distinct samples or whether the same sample was measured repeatedly
☐	☒	The statistical test(s) used AND whether they are one- or two-sided<br>*Only common tests should be described solely by name; describe more complex techniques in the Methods section.*
☐	☒	A description of all covariates tested
☐	☒	A description of any assumptions or corrections, such as tests of normality and adjustment for multiple comparisons
☐	☒	A full description of the statistical parameters including central tendency (e.g. means) or other basic estimates (e.g. regression coefficient) AND variation (e.g. standard deviation) or associated estimates of uncertainty (e.g. confidence intervals)
☐	☒	For null hypothesis testing, the test statistic (e.g. *F*, *t*, *r*) with confidence intervals, effect sizes, degrees of freedom and *P* value noted<br>*Give P values as exact values whenever suitable.*
☒	☐	For Bayesian analysis, information on the choice of priors and Markov chain Monte Carlo settings
☒	☐	For hierarchical and complex designs, identification of the appropriate level for tests and full reporting of outcomes
☒	☐	Estimates of effect sizes (e.g. Cohen's *d*, Pearson's *r*), indicating how they were calculated

Our web collection on statistics for biologists contains articles on many of the points above.

Software and code

Policy information about availability of computer code

Data collection	Simulated data were produced using the ART simulation tools (version MountRainier). All other data used in the study have been previously published and are publicly available; no software was used to obtain the remaining data.
Data analysis	Code related to the Meta-MARC software release can be found in the public repository on GitHub: https://github.com/lakinsm/meta-marc. Custom scripts and other data analytic code used in the manuscript can be found with descriptions in the public Meta-MARC publication repository on GitHub: https://github.com/lakinsm/meta-marc-publication. The remaining software used in the study include the following:<br><br>HMMER (3.1b2)<br>BLAST+ (2.4.0)<br>USEARCH (9.2.64)<br>Trimmomatic (0.36)<br>Burrows-Wheeler Aligner (0.7.13-r1126)<br>IDBA-UD (commit hash a1bafe6)<br>MetaGeneMark (3.26)<br>Resfams (1.2, full database)<br>R statistical programming language (3.3.2)

For manuscripts utilizing custom algorithms or software that are central to the research but not yet described in published literature, software must be made available to editors/reviewers. We strongly encourage code deposition in a community repository (e.g. GitHub). See the Nature Research guidelines for submitting code & software for further information.

Data

Policy information about availability of data

All manuscripts must include a data availability statement. This statement should provide the following information, where applicable:

- Accession codes, unique identifiers, or web links for publicly available datasets
- A list of figures that have associated raw data
- A description of any restrictions on data availability

The datasets used in this study are publicly available at https://megares.meglab.org and at the National Center for Biotechnology Information BioProject Accessions PRJNA215106, PRJNA244044, and PRJNA2924710. The data used in this study are available with no restrictions.

Field-specific reporting

Please select the one below that is the best fit for your research. If you are not sure, read the appropriate sections before making your selection.

☒ Life sciences ☐ Behavioural & social sciences ☐ Ecological, evolutionary & environmental sciences

For a reference copy of the document with all sections, see nature.com/documents/nr-reporting-summary-flat.pdf

Life sciences study design

All studies must disclose on these points even when the disclosure is negative.

Sample size	The data used in this study involve previously published high-throughput sequencing data and were not subjected to a sample size calculation for inclusion in this study. The data related to NCBI BioProject Accessions PRJNA215106, PRJNA244044, and SRP067931 had 169 samples, 219 samples, and 34 samples respectively.
Data exclusions	No data were excluded from the analysis.
Replication	The data generated in this study was done using code with seeded pseudo-random number generators where possible, and as such the results can be reproduced. The code used in the study is publicly available on GitHub as described above.
Randomization	For simulated data, leave-one-out cross-validation was performed that does not involve randomization. All other data were used as previously published with no additional manipulation to study design.
Blinding	Blinding was not relevant to this study as it was primarily descriptive of new bioinformatics methodology.

Reporting for specific materials, systems and methods

We require information from authors about some types of materials, experimental systems and methods used in many studies. Here, indicate whether each material, system or method listed is relevant to your study. If you are not sure if a list item applies to your research, read the appropriate section before selecting a response.

Materials & experimental systems

n/a	Involved in the study	
☒	☐	Antibodies
☒	☐	Eukaryotic cell lines
☒	☐	Palaeontology
☒	☐	Animals and other organisms
☒	☐	Human research participants
☒	☐	Clinical data

Methods

n/a	Involved in the study	
☒	☐	ChIP-seq
☒	☐	Flow cytometry
☒	☐	MRI-based neuroimaging
